# A approach of gastric conduit via the anterior of pulmonary hilum route during minimally invasive McKeown esophagectomy

**DOI:** 10.1186/s13019-024-02718-7

**Published:** 2024-04-16

**Authors:** Zhaoyang Yan, Xinjian Xu, Bin Guo, Pengzeng Wang, Linpeng Niu, Zhanjie Gao, Yusen Yuan, Fei Li, Ming He

**Affiliations:** Department of Thoracic Surgery, The Fourth Hospital of Hebei Medical University, Hebei Provincial Key Laboratory of Tumor Microenvironment and Drug Resistance, Shijiazhuang, Hebei Province China

**Keywords:** Esophagectomy, Gastric conduit, Anterior of the pulmonary hilum route

## Abstract

**Background:**

The gastric conduit is the most commonly used replacement organ for reconstruction after minimally invasive McKeown esophagectomy. Although the optimal route of gastric conduit remains controversial, the posterior mediastinal route is physiologically preferable but is not without disadvantages. Here, we report the safety and efficacy of a method of gastric conduit reconstruction via the anterior of the pulmonary hilum route.

**Methods:**

We have used the anterior of the pulmonary hilum route since 2021. This procedure involves pulling the gastric conduit up through a substernal tunnel between the right thoracic cavity and the abdominal cavity and passing it into the neck via the anterior of the pulmonary hilum route. In this retrospective study, we compared the clinical outcomes between 20 patients who underwent this procedure and 20 patients who underwent the posterior mediastinal route from 2021 to 2022.

**Results:**

No mortality was reported in either group. No significant differences were observed between the two groups in duration of surgery, blood loss, incidence of postoperative complications, and postoperative hospital stay. As a result of the anterior of the pulmonary hilum route, the primary tumor bed and lymph node drainage area were effectively bypassed, which facilitates postoperative adjuvant radiotherapy or chemoradiotherapy. The distance of the gastric conduit accompanying the airway was significantly shorter in the anterior of the pulmonary hilum route group.

**Conclusions:**

Our method is considered to be a safe and useful technique for the reconstruction of gastric conduit.

**Supplementary Information:**

The online version contains supplementary material available at 10.1186/s13019-024-02718-7.

## Background

Esophageal cancer (EC) is a highly aggressive malignancy that ranks seventh in incidence and sixth in mortality worldwide [1]. At present, surgery is the primary curative option for resectable EC [2]. McKeown esophagectomy is the most common operative approach for upper and middle thoracic EC and involves a laparotomy, right thoracotomy, and cervical anastomosis [3]. After esophagectomy, a gastric conduit formed by resection of the lesser curvature is the first choice of an esophageal substitute for reconstruction [4, 5]. In the McKeown procedure, esophageal reconstruction with the gastric conduit is usually performed via the posterior mediastinal route, which is considered the most physiological option [6]. Despite its advantages, this technique has some disadvantages. After esophagectomy, radiotherapy is an effective way to reduce regional recurrence [7]. However, radiotherapy’s side effects on the bypass conduit pose a significant concern for patients with the posterior mediastinal route. In addition, post-esophagectomy airway fistula, a severe morbidity associated with the posterior mediastinal reconstruction approach, is a life-threatening condition that can result in respiratory failure and septic shock and typically occurs with an anastomotic leak [8]. Accumulated digestive content between the gastric conduit and the tracheobronchial tree can lead to the development of an airway fistula [9]. Therefore, reducing the accompanying distance between the gastric conduit and the airway (trachea and bronchi) may decrease the incidence of respiratory-digestive fistula. Here, we report a method of gastric conduit reconstruction via the anterior of the pulmonary hilum route during minimally invasive McKeown esophagectomy, which can be performed to leave space for radiotherapy in the posterior mediastinum and reduce the accompanying distance between the gastric conduit and the airway.

## Methods

A total of 40 consecutive patients with upper and middle thoracic EC underwent minimally invasive McKeown esophagectomy at the Fourth Hospital of Hebei Medical University between January 2021 and September 2022. Gastric conduit reconstruction via the anterior of the pulmonary hilum route was performed in 20 patients, and gastric conduit reconstruction via the posterior mediastinal route was performed in the other 20 patients. General information about the patients, including age, gender, body mass index (BMI), and neoadjuvant chemotherapy, were recorded. The depth of tumor invasion and the status of lymph node metastasis were obtained from postoperative pathology reports. Patients were staged according to the eighth edition of the TNM classification [10]. Computed tomography scanning was performed one month after surgery. The distance of the gastric conduit accompanying with the airway (trachea and bronchi) was measured by postoperative computed tomography scanning.

### Surgical techniques

All patients underwent a thoracic-laparoscope-assisted McKeown esophagectomy with two-field lymph node dissection. The thoracoscopic phase was performed as previously described [11]. Briefly, the patient was placed in the left lateral decubitus position, and the observational incision was located at the seventh intercostal space on the posterior axillary line. The main operating port was located at the fourth intercostal space on the posterior axillary line and another operating port was located at the sixth intercostal space on the subscapular angle line. The assisted port was placed in the ninth intercostal space on the subscapular angle line. The para-esophageal and recurrent laryngeal nerve lymph nodes were dissected following complete circumferential mobilization of the thoracic esophagus.

In the second step, the patient was placed in a supine position. During the laparoscopic procedure, five trocars were inserted in the upper abdominal quadrant. The mobilization of the stomach was initiated by dividing the hepatogastric ligament. Next, the left gastric artery was separated and ligated at two ends using hemlock clips. The stomach was fully mobilized along the greater curvature from the spleen. In this procedure, the right gastroepiploic vascular arcade was preserved. A small incision was then made along the anterior margin of the sternocleidomastoideus muscle on the left side of the neck. Following appropriate exposure, the cervical esophagus was dissected (Figure S1A) and delivered through the hiatus into the abdomen (Figure S1B). Subsequently, the esophageal hiatus was routinely sutured (Figure S1C, D).

**Fig. 1:**
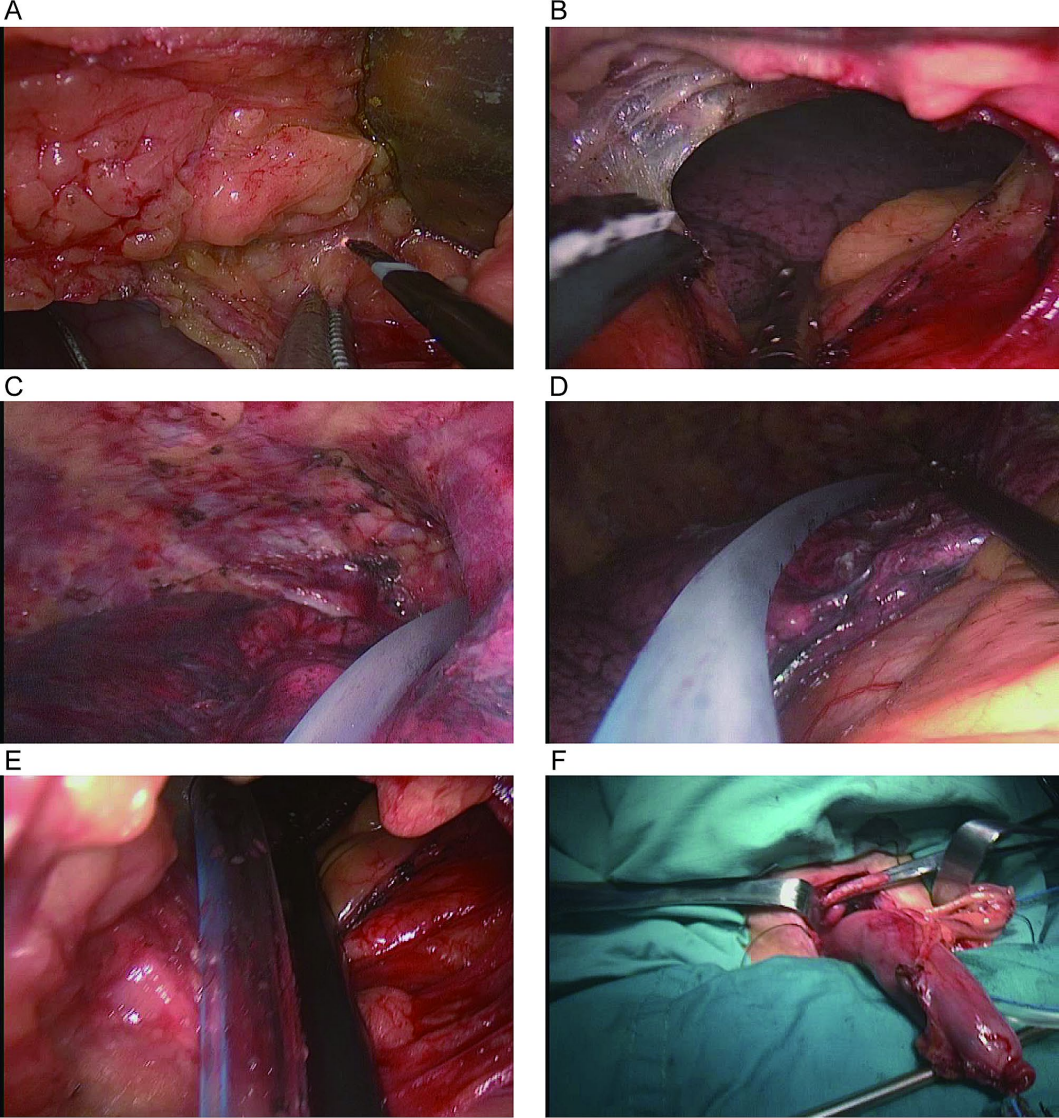
Intraoperative images of pulling up the gastric conduit via the anterior of pulmonary hilum route to the right thoracic cavity. (**A**, **B**) Create a substernal tunnel between the right thoracic cavity and the abdominal cavity at the inferior xiphoid process. (**C** - **E**) Connect the abdomen and neck through the anterior of the pulmonary hilum route using a nasogastric tube as a guide. (**F**) Cervical anastomosis

**Fig. 2:**
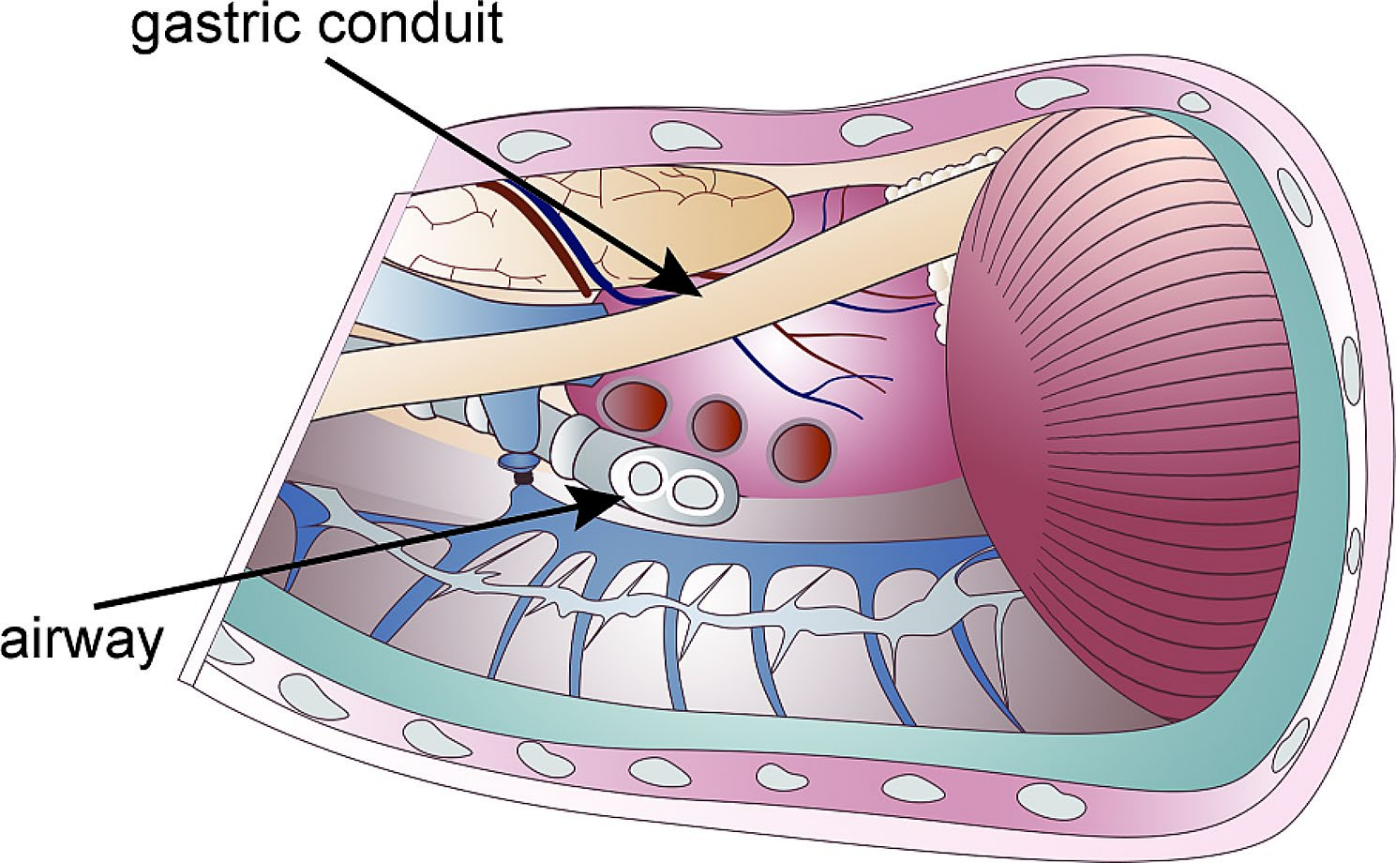
Schematic illustration of the key procedure in surgery

Next, a 5-cm incision was made just below the xiphoid (Figure S1E). The stomach was pulled from the abdominal cavity and a linear stapler was used to make the gastric conduit (Figure S1F). The width of the gastric conduit is 3 to 4 cm. Blunt dissection was performed at the inferior xiphoid process, and a substernal tunnel between the right thoracic cavity and the abdominal cavity was created (Fig. 1A and B). A 16-Fr nasogastric tube was used as a guide for connecting the abdomen and neck through the anterior of the pulmonary hilum route (Fig. 1C and E). The gastric conduit was pulled out of the neck incision, and the final step was cervical anastomosis (Fig. 1F). The overall schematic representation is shown in Fig. 2.

### Statistical analysis

Student’s t-test was applied to compare continuous variables and the chi-square test or Fisher’s exact test was applied to analyze categorical variables. All of the analyses were performed using STATA 15.0 (StataCorp Texas, USA). For all analyses, a *P*-value < 0.05 was considered significant.

## Results

The clinicopathological characteristics of patients are summarized in Table 1. The two groups were well matched for baseline characteristics including age, gender, tumor location, BMI, neoadjuvant chemotherapy, and staging.

**Table 1:** Basic clinical characteristics of the patients

Characteristics	Anterior of the pulmonary hilum route group (*n* = 20)	Posterior mediastinal route group (*n* = 20)	P value
Age (years)	64.4 ± 4.8	61.7 ± 4.4	0.10
Gender			0.72
Male	16	14	
Female	4	6	
Tumor location			0.73
Upper	5	7	
Middle	15	13	
BMI (kg/m^2^)	22.7 ± 2.6	22.3 ± 2.7	0.72
Neoadjuvant chemotherapy			0.75
Yes	9	11	
No	11	9	
Depth of tumor invasion			0.89
T1	6	5	
T2	1	3	
T3	12	11	
T4	1	1	
Lymph node metastasis			0.47
N0	11	10	
N1	5	8	
N2	4	2	
TNM stage			0.84
I	5	4	
II	5	7	
III	9	9	
IV	1	0	

**Fig. 3:**
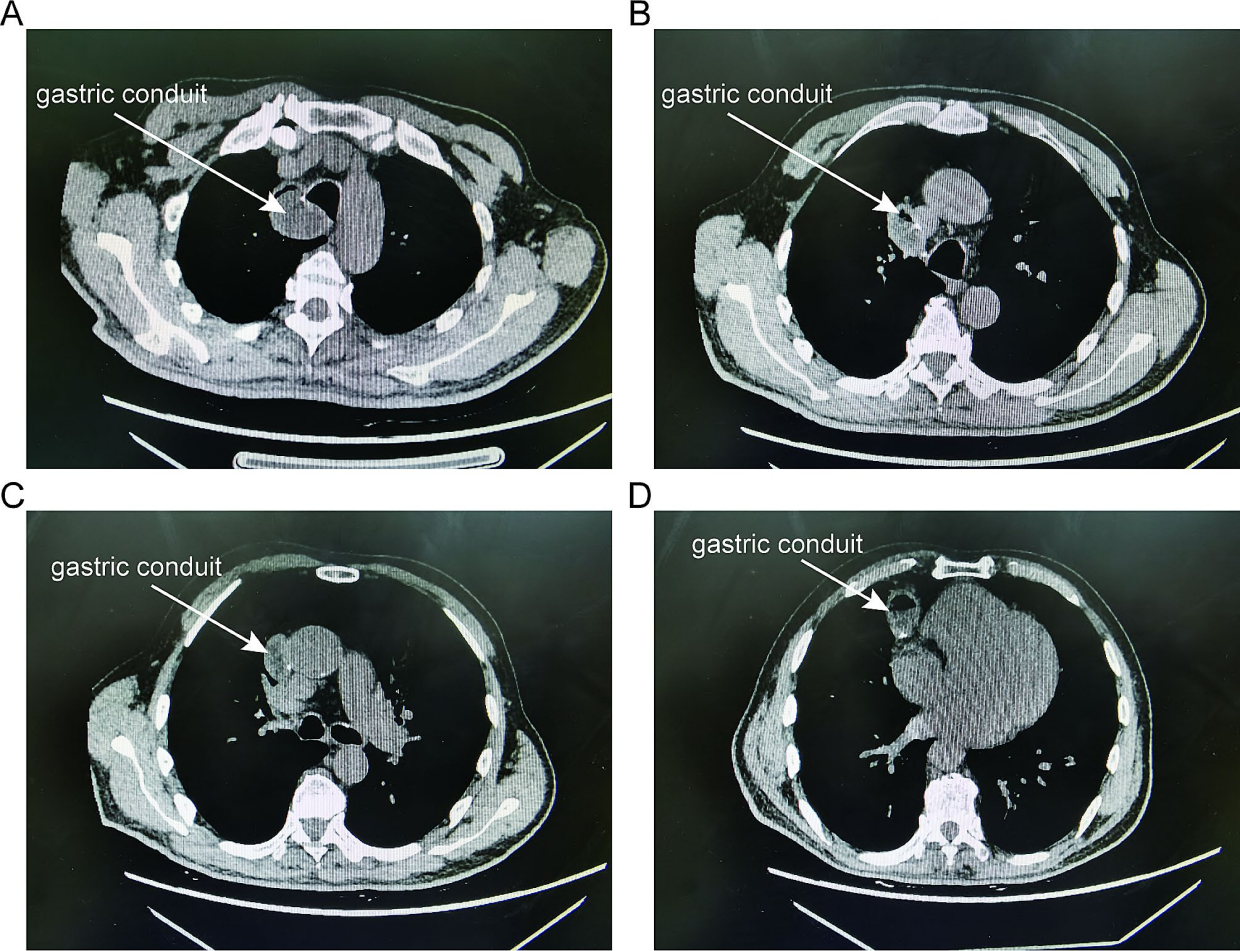
Radiographic images after surgery. (**A**-**D**) Axial images of postoperative chest computed tomography of the patient. The gastric conduit is passed across the anterior of the pulmonary hilum

There was no significant difference between the two groups in the duration of surgery, blood loss, the incidence of postoperative complications, and postoperative hospital stay. No mortality was observed in either group. The distance of the gastric conduit accompanying the airway was significantly shorter in the anterior of the pulmonary hilum route group (*P* < 0.01) (Table 2). Representative radiographic pictures of the anterior of the pulmonary hilum route group are shown in Fig. 3.

**Table 2:** Surgical results of the patients

Characteristics	Anterior of the pulmonary hilum route group (*n* = 20)	Posterior mediastinal route group (*n* = 20)	P value
Operation time (min)	452.0 ± 66.6	429.2 ± 56.0	0.23
Blood loss (ml)	212.5 ± 60.1	203.8 ± 53.5	0.61
Morbidity			
Respiratory complications	2	2	1.0
Anastomotic leakage	2	3	1.0
Hoarseness	4	5	1.0
Mortality	0	0	
Postoperative hospital stay (days)	15.3 ± 6.1	15.4 ± 6.2	1.0
The distance of the gastric conduit accompanying the airway (mm)	66.9 ± 13.1	143.5 ± 16.6	< 0.01

## Discussion

Minimally invasive McKeown esophagectomy is becoming increasingly popular because it is associated with a low likelihood of trauma and rapid recovery [12]. However, esophageal reconstruction remains a challenging aspect of the procedure, and there is a lack of consensus on the optimal route for reconstruction in patients who require an esophagectomy [13, 14]. The posterior mediastinal route is preferred by some surgeons because it is relatively shorter and is associated with fewer cardiopulmonary complications and anastomotic leaks [15, 16]. Nevertheless, this method has some limitations. For patients with advanced esophageal cancer following R1 or R2 resection, postoperative adjuvant radiotherapy is preferred [17]. For patients with R0 resection, there is a still high rate of tumor recurrence. Thus, adjuvant radiotherapy is an effective modality to reduce the likelihood of local recurrence [18]. Due to the occupation of the position of the esophageal bed, esophageal reconstruction via the posterior mediastinal route is unfavorable to the development of radiotherapy plans [19]. In addition, when anastomotic leakage occurs, the digestive content accumulated in the esophageal bed may not be adequately drained. A longer distance of the gastric conduit accompanying the airway may be associated with an increased likelihood of airway fistula.

In our method, the gastric conduit was pulled up from the subxiphoid and bypassed the anterior of the pulmonary hilum to reach the neck. The process of creating a tunnel under the xiphoid was associated with minimal trauma. We showed that the duration of surgery, intraoperative blood loss, intraoperative complications, postoperative complications, and length of hospital stay were not significantly different between the two groups. Moreover, in our approach, the gastric conduit was not located in the posterior mediastinum, meaning that radiotherapy of the esophageal bed was unaffected. Another advantage of our technique was that the gastric conduit was close to the trachea only at the neck. When anastomotic leakage occurred, the digestive content could therefore be easily drained, resulting in a reduction of the incidence of airway fistula. Even where airway fistula did occur, the anastomotic leakage and airway leakage were not directly connected nor on the same horizontal plane, making this complication easier to treat.

The retrosternal route is an alternative surgical approach to avoid the effects of irradiation on the gastric conduit in the adjuvant radiotherapy process and decrease the distance of the gastric conduit accompanying the airway [20, 21]. However, the creation of the retrosternal tunnel may increase surgical trauma. Adequate blood flow plays an important role in the healing process of the anastomotic site, and the narrow space at the sternal stalk may compress the gastric conduit and affect the blood supply of the anastomosis. Some surgeons also consider the tight angulation of the thoracic inlet to increase the risk of anastomotic leakage in patients who undergo retrosternal reconstruction [22].

The present study also has some limitations. First, it was a small-scale, non-randomized, single-institute retrospective study. In addition, the observation time was relatively short, preventing the examination of long-term postoperative functional outcomes.

## Conclusions

In summary, gastric conduit reconstruction via the anterior of the pulmonary hilum route during minimally invasive McKeown esophagectomy is a safe and useful technique and can be considered suitable for widespread application in clinical practice.

### Electronic supplementary material

Below is the link to the electronic supplementary material.

Supplementary Material 1

## Data Availability

The datasets used and analyzed during the current study are available from the corresponding author on reasonable request.
