# Supplementary material for: A approach of gastric conduit via the anterior of pulmonary hilum route during minimally invasive McKeown esophagectomy
Source: J Cardiothorac Surg. 2024 Apr 16;19:232. doi: 10.1186/s13019-024-02718-7 (PMC11020892; doi:10.1186/s13019-024-02718-7)

**Figure S1** Intraoperative images. (A) Dissect the cervical esophagus. (B) Pull the esophagus down to the abdominal cavity through esophageal hiatus. (C, D) Close the esophageal hiatus. (E) Make a small midline upper abdominal incision. (F) The gastric conduit.


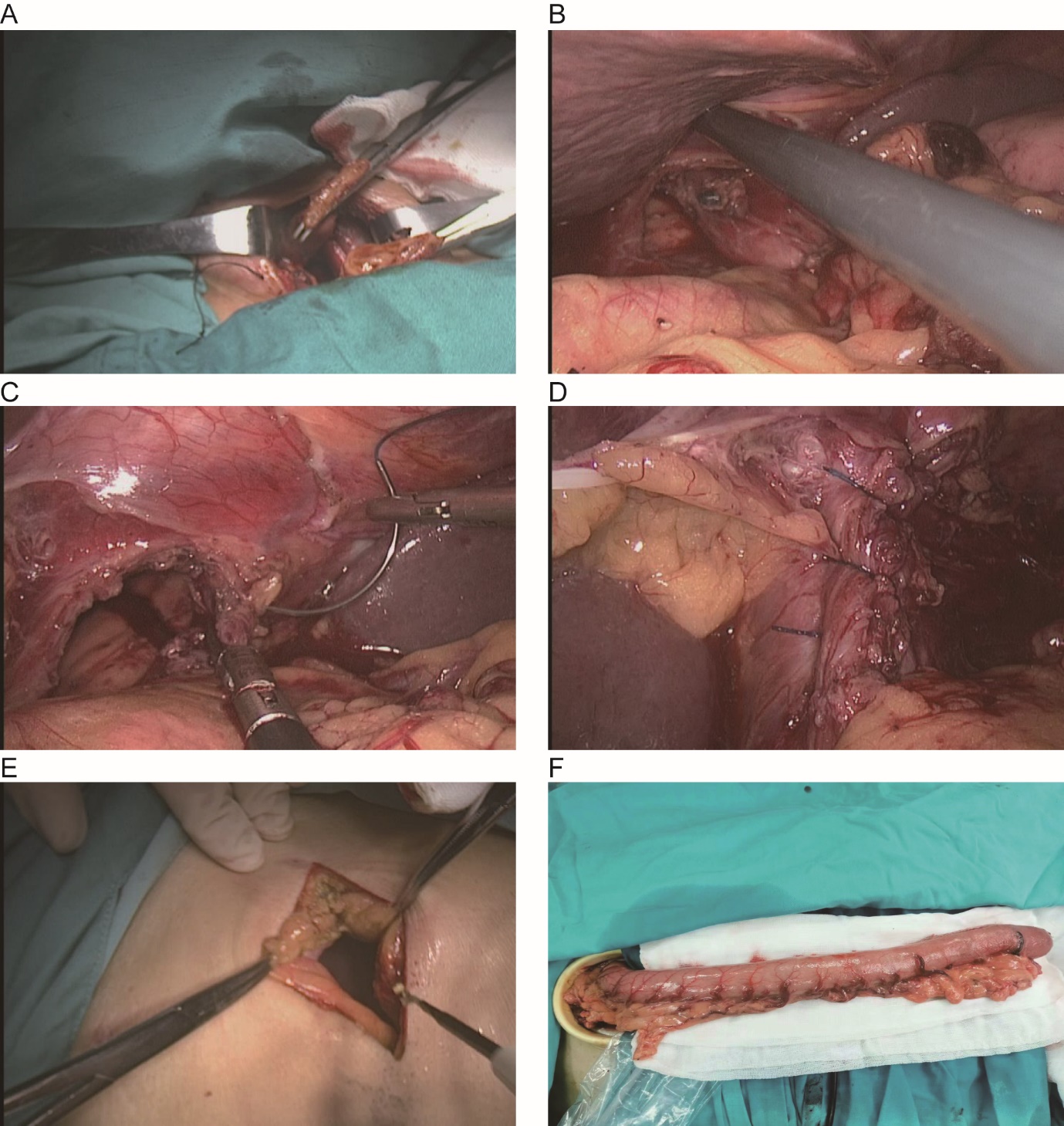

Supplement: Supplementary file 1 — Supplementary Material 1 [file 13019_2024_2718_MOESM1_ESM.docx]
